# Effects of *Magnolia denudata* extract on quality and storage characteristics of emulsified chicken sausage

**DOI:** 10.1007/s44463-025-00026-9

**Published:** 2026-06-10

**Authors:** Beobmo Ku, Minjun Kim, Jiwoo Kang, Hyodong Han, Gyutae Park, Sol-Hee Lee, Jungseok Choi

**Affiliations:** Department of Animal Science, Chungbuk National University, Cheongju, Chungcheongbuk-do 28644 Korea

**Keywords:** Magnolia denudata, Quality properties, Storage stability, Oxidation

## Abstract

This study aimed to evaluate the effects of *Magnolia denudata* extract (MDE) on the quality and storage stability of chicken breast sausages and to assess its potential as a natural alternative to synthetic antioxidants. MDE exhibited an antioxidant capacity of 44.48%, total polyphenol content of 132.91 mg GAE/g, and total flavonoid content of 42.44 mg NE/g. Chicken breast sausages were prepared with MDE at concentrations of 0.1% (T1), 0.15% (T2), and 0.2% (T3), and their quality characteristics were compared with a control group without any added extract. The analyzed parameters included proximate composition, color, cooking yield, water-holding capacity, sensory evaluation, and changes during storage, including pH, thiobarbituric acid reactive substance (TBARS), and volatile basic nitrogen (VBN). The results showed that increasing concentrations of MDE increased moisture content while reducing protein content in the sausages (*p* < 0.05). Cooking yield and water-holding capacity were significantly higher in the extract-treated groups compared to the control (*p* < 0.05). During storage, sausages with MDE exhibited less variation in pH compared to the control, with the 0.2% treatment group showing the most stable pH values. TBARS was significantly inhibited at all extract concentrations, with the 0.2% group showing the slowest increase (*p* < 0.05). Furthermore, the 0.2% extract group significantly reduced VBN content (*p* < 0.05), indicating better protein preservation and spoilage prevention. These findings suggest that the addition of 0.2% *Magnolia denudata* extract effectively enhances cooking properties and storage stability in chicken breast sausages, highlighting its potential as a safe and natural antioxidant alternative for improving processed meat quality.

## Introduction

The consumption of meat products in Korea has steadily increased over the past decade, rising from approximately 45.4 kg per person in 2018 to 50.6 kg in 2022, and is projected to reach nearly 60.0 kg by 2027 (Euromonitor, 2025). Additionally, as consumer interest in health and well-being grows, the meat processing industry is continuously developing various products using natural ingredients to meet consumer satisfaction and demands (Park et al., 2016). Synthetic antioxidants can be added to meat products to enhance storage stability. They play a role in stabilizing free radicals to inhibit lipid oxidation (Cho et al., 2006a) and delaying spoilage by suppressing microbial growth. However, synthetic antioxidants such as butylated hydroxyanisole (BHA) and butylated hydroxytoluene (BHT) have raised safety concerns in recent years. BHA has been classified by the U.S. National Toxicology Program as reasonably anticipated to be a human carcinogen (National Toxicology Program, 2021). Additionally, network toxicology analyses suggest potential hepatotoxic, nephrotoxic, and neurotoxic effects associated with both BHA and BHT (Ren et al., 2025). Consequently, there is a need for research on safer antioxidants. Efforts are actively being made to develop food ingredients using natural plant-based materials containing bioactive compounds, such as buckwheat, green tea, and licorice (Nam, 2024; Kim et al., 2002; Cho et al., 2006b).

*Magnolia denudata*, a deciduous shrub belonging to the family *Magnoliaceae*, contains various polyphenols and flavonoids in its flowers. These polyphenols and flavonoids are known for their antioxidant properties (Yoon, 2014). Additionally, *Magnolia denudata* contains various phenolic compounds, such as rutin, chlorogenic acid, magnolol, and honokiol (Park et al., 2018; Hyeon et al., 2021; Cristea et al., 2024), and exhibit antibacterial (Kim et al., 2015a, 2015b), anti-inflammatory (Mottaghi & Abbaszadeh, 2022), and antioxidant activities, indicating a high potential of *Magnolia denudata* for application in various food industries. Although numerous studies have demonstrated the antioxidant and antimicrobial activities of *Magnolia denudata*, research on its application in meat products to evaluate storage stability remains limited. Furthermore, no study has simultaneously assessed the physicochemical characteristics, oxidative stability, and sensory properties of emulsified chicken sausages. Based on its known antioxidant effects, we hypothesized that *Magnolia denudata* extract would inhibit lipid and protein oxidation during refrigerated storage and improving water-holding capacity and cooking yield. The ultimate goal of this study is to confirm the potential of using *Magnolia denudata* extract as a natural antioxidant to replace synthetic antioxidants.

## Materials and methods

### Experimental materials and Preparation of freeze-dried *Magnolia denudata* extract

**Fig. 1:**
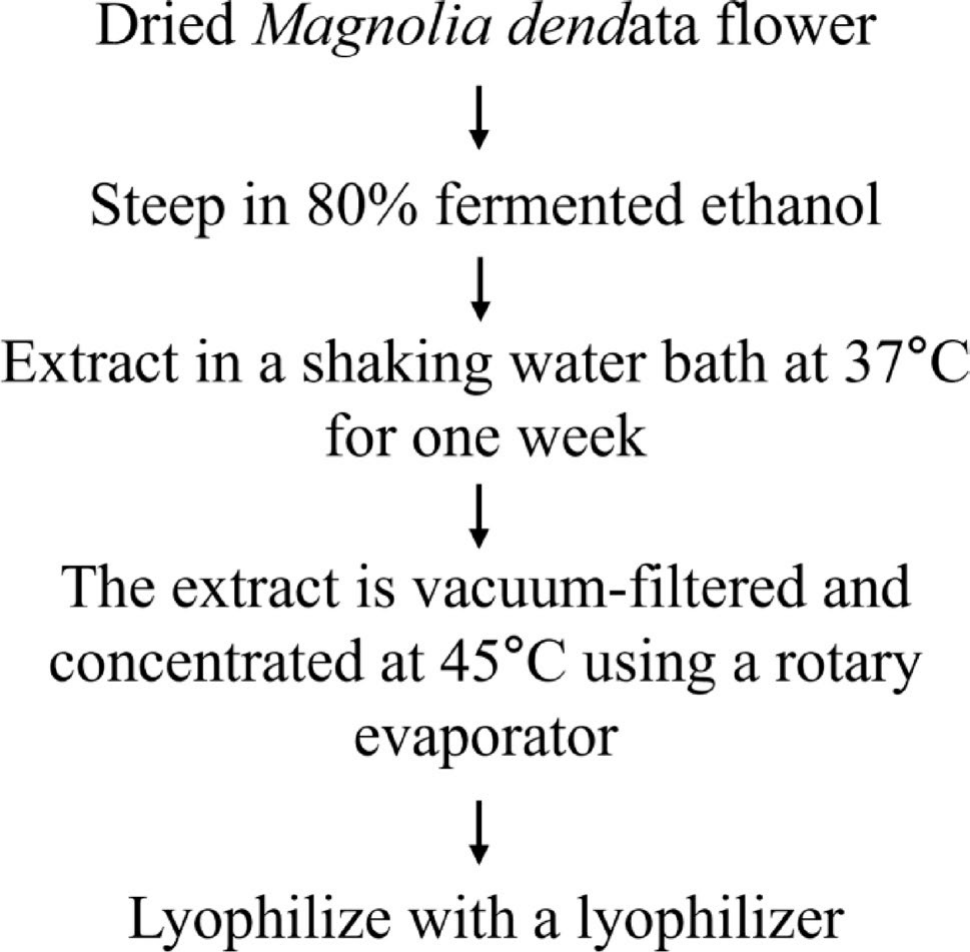
*Magnolia denudate* extraction procedure

Fully bloomed *Magnolia denudata* flower samples were collected in April 2024 from ornamental trees planted on the campus of Chungbuk National University. Collected flowers were washed immediately after harvest and dried at room temperature (25 °C) with good ventilation. The *Magnolia denudata* extract manufacturing method is shown in Fig. 1. To prepare the extract, an 80% ethanol solution diluted with distilled water at a ratio of 1:4 was added to dried flowers. The mixture was subjected to extraction for one week in a shaking water bath (Biofree, Korea) at 37 °C. The ethanol concentration was set based on the study (Lee et al., 2013). The extract was filtered under reduced pressure and concentrated using a rotary vacuum evaporator (EYELA, Japan) at 45 °C. The concentrated extract was freeze-dried using a freeze dryer (Bondiro, Korea) at −40 °C for 12 h and then stored frozen for further experiments. The extraction yield, calculated as the weight of the freeze-dried extract relative to the dried flower weight, was 5.33 ± 0.15% (w/w), based on three independent extractions. The coefficient of variation (CV) was less than 2%.

### Preparation of emulsion-type sausages

Chicken sausages with *Magnolia denudata* extract powder added were prepared as described in Table 1. All sausages had the same amount of ground chicken breast, pork backfat, ice, salt and sugar. Two control groups were used, including a negative control (NC) group without additives and a positive control (PC) group added with 0.1% ascorbic acid. Treatment groups were added with 0.1% (T1), 0.15% (T2), and 0.2% (T3) *cc*. The emulsified mixture was filled into 50 mL conical tubes using a filling gun and heated in a water bath at 80 °C for 30 min. Sausages were then air-cooled at room temperature (10 °C) for 20 min. These prepared sausages were stored under refrigerated conditions (4 °C) for two weeks and used for subsequent experiments.

**Table 1:** Formulation of chicken sausages with various ratio of *Magnolia denudata* extract

Ingredients (%)		PC	NC	T1	T2	T3
Main	Meat	60	60	60	60	60
	Fat	20	20	20	20	20
	Ice	20	20	20	20	20
Additive	Salt	1.2	1.2	1.2	1.2	1.2
	Sugar	1	1	1	1	1
	Ascorbic acid	0.1	-	-	-	-
	Magnolia leaf extract	-	-	0.1	0.15	0.2

### Measurement of total phenol content (TPC)

TPC was measured using a modified method of study (Lee et al., 2020b; Choi et al., 2022). Briefly, a mixture of 40 µL of *Magnolia denudata* extract supernatant and 80 µL of 2 N Folin-Ciocalteu reagent was prepared. After 3 min, 800 µL of Na₂CO₃ was added and the mixture was vortexed. The reaction was carried out in the dark at 37 °C for 30 min. Absorbance was measured at 765 nm using a multi-mode microplate reader (SpectraMax iD3, Molecular Devices, San Jose, CA, USA). A standard curve was prepared using gallic acid. Results are expressed as mg GAE/g (GAE: gallic acid equivalents). All measurements were performed in triplicate for each treatment group (*n* = 3).

### Measurement of total flavonoid content (TFC)

TFC was determined following the method described by (Choi et al., 2022). Briefly, a mixture of 100 µL of *Magnolia denudata* extract supernatant, 1 mL of diethylene glycol, and 100 µL of 1 N NaOH was vortexed and incubated in the dark at 37 °C for 1 h. Absorbance was measured at 420 nm using a multi-mode microplate reader. A standard curve was prepared using naringin. Results are expressed as mg NE/g (NE: naringin equivalents). All measurements were performed in triplicate for each treatment group (*n* = 3).

### 2,2-Diphenyl-1-picrylhydrazy (DPPH) radical scavenging activity

The DPPH radical scavenging activity was measured using a modified method of study (Choe et al., 2014). Briefly, 1 mL of *Magnolia denudata* extract supernatant was mixed with 1 mL of ethanol and 2 mL of 0.2 mM DPPH solution and then vortexed. The mixture was incubated in the dark at room temperature for 30 min and absorbance was measured at 517 nm using a multi-mode microplate reader. All measurements were performed in triplicate for each treatment group (*n* = 3). The DPPH scavenging activity was calculated using the following formula:

$$\:Scavenging\:Activity\:\left(\%\right)=\:\frac{a-b}{a}\times\:100$$

where a was the absorbance of the control and b was the absorbance of the sample.

### Proximate composition analysis

Proximate composition was analyzed following the AOAC (2007) guidelines. Moisture content was determined by oven drying at 105 °C. Crude protein content was analyzed using the DUMAS method, crude fat content using the Folch method, and ash content using direct incineration at 550 °C. All measurements were performed in triplicate for each treatment group (*n* = 3).

### pH measurement

To measure pH, 5 g of the sample was homogenized with 45 mL of distilled water using a homogenizer (Stomacher 400 Circulator, Seward, UK) for 30 s. The pH was measured using a pH meter (Orion Star™ A211, Thermo Scientific, UK), calibrated with pH 4, 7, and 10 buffer solutions. All measurements were performed in triplicate for each treatment group (*n* = 3).

### Color measurement

The inner surface of cooked sausages was measured for color using a colorimeter (CM-26d, Konica Minolta, Japan). The lightness (CIE L*), redness (CIE a*), and yellowness (CIE b*) were measured three times per sample. Calibration was performed using a calibration plate with the following values: L* = 99.41, a* = −0.13, b* = −0.11. All measurements were performed in triplicate for each treatment group (*n* = 3).

### Cooking yield measurement

The cooking yield of sausages containing freeze-dried *Magnolia denudata* extract powder was calculated by measuring the weight before and after cooking. All measurements were performed in triplicate for each treatment group (*n* = 3). Cooking yield was expressed as a percentage using the following formula

$$\:Cooking\:yield\:\left(\%\right)=\:\frac{Weight\:after\:cooking}{Weight\:before\:cooking}\times\:100$$.

### Water holding capacity (WHC)

0.5 g sample was placed in an upper filter of a centrifuge tube, weighed, and heated in a water bath at 80 °C for 20 min. The sample was centrifuged at 800 ×g for 10 min, and the filter tube was reweighed to determine the water retained. All measurements were performed in triplicate for each treatment group (*n* = 3).

### Thiobarbituric acid reactive substances (TBARS) measurement

TBARS were measured by weighing 5 g of the sample into a homogenizer cup, adding 15 mL of distilled water and 100 µL of 7.2% BHT. The mixture was homogenized at 11,200 ×g for 1 min and filtered using Whatman No. 2 filter paper. 1mL of the filtrate was mixed with 2 mL of TBA/TCA reagent (20 mM TBA in 15% TCA), heated at 90 °C for 30 min, cooled for 10 min, and centrifuged at 3,000 rpm for 15 min. Absorbance was measured at 532 nm, and TBARS were expressed as mg malonaldehyde (MDA) per kg of the sample. All measurements were performed in triplicate for each treatment group (*n* = 3).

### Volatile basic nitrogen (VBN) measurement

VBN was measured by homogenizing 5 g of the sample with 45 mL of distilled water at 10,000 rpm for 60 s. The homogenate was filtered using Whatman No. 2 filter paper, and 1 mL of the filtrate was placed in the outer chamber of a Conway unit. The inner chamber contained 1 mL of 0.01 N boric acid solution and 50 µL of a mixed indicator (0.066% methyl red + 0.066% bromocresol green). The lid was sealed with vaseline, and 1 mL of 50% K₂CO₃ was added to the outer chamber. After horizontal shaking, the mixture was incubated at 37 °C for 120 min. The inner chamber solution was titrated with 0.02 N H₂SO₄, and the results were expressed as mg VBN per 100 g of the sample. All measurements were performed in triplicate for each treatment group (*n* = 3).

### Sensory evaluation

A total of 10 trained panelists (6 males and 4 females, aged 24–30 years) with prior experience in meat sensory evaluation participated in the study. Sensory evaluation was conducted in individual booths under white fluorescent lighting at 25 °C, using a 10-point hedonic scale. Attributes evaluated included off-order, appearance, tenderness, juiciness, flavor, saltiness, bitterness, and overall preference. The scoring criteria were as follows: off-ordor (1 = minimal, 10 = extreme), appearance (1 = very poor, 10 = excellent), tenderness (1 = very soft, 10 = very firm), juiciness (1 = very dry, 10 = very juicy), flavor (1 = very poor, 10 = excellent), saltiness (1 = weak, 10 = strong), bitterness (1 = weak, 10 = strong), and overall preference (1 = very poor, 10 = excellent). The study complied with ethical guidelines (IRB Approval No. CBNU-202302-HR-0017).

### Statistical analysis

All statistical analyses were conducted using SPSS software (version 28.0, SPSS Inc., Chicago, IL, USA). One-way analysis of variance (ANOVA) was performed to assess statistical significance, followed by Duncan’s test for post hoc analysis at a significance level of *p* < 0.05.

## Result and discussion

### DPPH radical scavenging activity and phenolic compound content of *Magnolia denudate* extract

Table 2 shows DPPH radical scavenging activity, TPC, and TFC results. In this study, an experiment was conducted to evaluate the antioxidant activity of *magnolia denudate* extract by comparing it with ascorbic acid, a well-known antioxidant. DPPH is a stable purple radical that changes its color to yellow when it reacts with an antioxidant, allowing for the measurement of antioxidant activity (Brand-Williams 1999). The *Magnolia denudata* extract showed an average DPPH radical scavenging rate of 44.48%, similar to or higher than antioxidant activities of edible spring flowers such as Forsythia (40.4%), Rhododendron (34.5%), and Cherry Blossom (43.3%) (Kim et al., 2014). These results suggest that antioxidants, including polyphenols and flavonoids, in the *Magnolia denudata* extract can positively influence its radical scavenging ability. Its antioxidant activity is superior to those of other edible spring flowers. Total polyphenol and flavonoid contents of the *Magnolia denudata* were analyzed using gallic acid and naringin as standard substances. Its total polyphenol content was measured at 132.91 (mg GAE/g), similar to the 143.9 (mg GAE/g) found in a study (Yoon, 2014). Its total flavonoid content was 42.44 (mg NE/g), similar to the 56.93 (mg CE/g) found in a study (Nho et al., 2009). Previous studies have reported that plant extracts with antioxidant activity can enhance storage stability of meat products (Shah et al., 2014; Falowo et al., 2014). Based on this evidence, the antioxidant activity of *Magnolia denudata* extract, attributable to its polyphenol and flavonoid contents, is also expected to contribute to improving shelf life.

**Table 2:** 1-picrylhydrazy (DPPH), total polyphenol contents (TPC), and total flavonoid contents (TFC) of *Magnolia denudate* extract

Traits^1^	Magnolia denudate Extract
DPPH (%)	44.48 ± 0.01
TPC (mg GAE/g)	132.91 ± 0.97
TFC (mg NE/g)	42.44 ± 1.27

^1^GAE: gallic acid equivalents, NE: naringin acid equivalents

### Proximate composition analysis

Table 3 shows moisture content, crude protein content, crude fat content, and ash content of sausages based on the amount of *Magnolia denudata* extract added. In terms of moisture content, treatment groups showed higher values than the negative control (NC) and positive control (PC). There was a trend of increasing moisture content as the concentration of *Magnolia denudata* extract increased from 0.1% to 0.2%. The T1, T2 and T3 showed significantly higher moisture content than both NC and PC (both *p* < 0.05). The increase in moisture content might be due to insoluble dietary fibers. According to study (Boulos, 2000), a higher content of cellulose can increase moisture binding capacity. The protein content was the highest in the NC group. Treatment groups showed significantly (*p* < 0.05) lower protein contents than the NC group when concentrations of the *Magnolia denudata* extract added to treatment groups were increased. Hong et al. (2020) has reported that when mustard powder is added to meat products, the increase in moisture content due to dietary fiber in mustard powder can lead to a relative decrease in protein content, similar to results of the current study. The fat content was the lowest in the T3 group, followed by NC and T1, while PC and T2 had significantly higher fat content (*p* < 0.05). The lower fat content observed in the T3 group can be attributed to its higher moisture content, which likely reduced the relative proportion of fat in the proximate composition due to a dilution effect. The higher ash content in the NC group was thought to be due to natural mineral contents in raw materials. The ash content in processed meat products can vary depending on mineral contents in raw materials. Minerals in meat bones or muscles can significantly affect ash contents in sausages. On the other hand, although additives such as *Magnolia denudata* extract could influence ash content, no significant difference in ash content was observed in *Magnolia denudata* extract-treated groups (T1, T2, T3). This suggests that the *Magnolia denudata* extract might have had minimal impact on ash content due to dilution of certain components or specific properties of compounds.

**Table 3:** Proximate composition of chicken breast sausage formulated with various levels of *Magnolia denudate* extract

Traits(%)	NC	PC	T1	T2	T3
Moisture	64.7 ± 1.73^b^	64.6 ± 1.47^b^	67.7 ± 1.57^a^	67.2 **±** 0.16^a^	70.1 ± 0.55^a^
Crude protein	17.93 ± 0.18^a^	16.50 ± 0.45^b^	15.92 ± 0.17^b^	16.57 ± 0.17^b^	16.02 ± 0.20^b^
Crude fat	10.06 ± 0.13^b^	10.58 ± 0.05^a^	10.09 ± 0.34^b^	10.77 ± 0.03^a^	9.32 ± 0.02^c^
Crude ash	1.8 ± 0.17^a^	1.5 ± 0.12^b^	1.4 ± 0.07^b^	1.5 ± 0.04^b^	1.5 ± 0.09^b^

NC, no addition; PC, Ascorbic acid 0.1%; T1, *Magnolia denudate* extract 0.1%; T2, *Magnolia denudate* extract 0.15%; T3, *Magnolia denudate* extract 0.2%

Means in the same row (a–c) with different letters are significantly different (*p* < 0.05)

All values are mean ± standard error

### Color

Table 4 shows color values based on the level of *Magnolia denudata* extract added. The color of sausages can significantly affect consumer purchase decisions. It plays a key role in assessing freshness and quality of sausages (Resurreccion, 2004). In terms of lightness, the NC group showed the highest value, which was significantly different from other groups (*P* < 0.05), while T2 showed the lowest lightness value (*P* < 0.05). In terms of redness, T3 showed the highest value, significantly higher than other groups (*P* < 0.05), while T1 showed the lowest value (*P* < 0.05). As the concentration of *Magnolia denudata* extract increased, redness also increased, with the highest value observed for the group added with 2% *Magnolia denudata* extract. This is consistent with reports showing that antioxidants like polyphenols and flavonoids as major pigments in plants can display a wide range of colors from yellow to red, with antioxidant capacity having a correlation with color (Kim et al., 2015a). The yellowness of the NC group was the lowest, showing significantly lower than T1, T2 and the PC group (*p* < 0.05), but no significant difference was observed compared to T3 (*p* > 0.05). The higher yellowness observed in the PC group may also be partly attributed to its higher fat content, as fat can impart a light yellow hue and enhance light scattering, thereby increasing yellowness. The elevated yellowness observed in T1 and T2 may be due to the inherent brownish color of the *Magnolia denudata* extract itself, which can naturally increase the yellowness in meat products. In contrast, T3 showed no significant difference compared to NC, which can be explained by the dilution of pigment concentration due to its higher moisture content. According to Hughes et al. (2014), increased water in muscle can alter light scattering properties and reduce the perceived intensity of meat color. This optical effect, together with pigment dilution, may have contributed to the lower yellowness observed in T3.

**Table 4:** Color of chicken breast sausage formulated with various levels of *Magnolia denudate* extract

Traits(%)	NC	PC	T1	T2	T3
CIE L^*^	85.27 ± 0.58^a^	83.39 ± 0.35^bc^	83.55 ± 0.30^b^	82.26 ± 1.35^c^	84.54 ± 1.15^ab^
CIE a*	−0.02 ± 0.15^abc^	−0.068 ± 0.08^bc^	−0.18 ± 0.13^c^	0.05 ± 0.08^ab^	0.14 ± 0.21^a^
CIE b*	15.67 ± 0.42^b^	16.29 ± 0.46^a^	16.56 ± 0.25^a^	16.41 ± 0.36^a^	16.12 ± 0.35^ab^

NC, no addition; PC, Ascorbic acid 0.1%; T1, *Magnolia denudate* extract 0.1%; T2, *Magnolia denudate* extract 0.15%; T3, *Magnolia denudate* extract 0.2% CIE L*, lightness; CIE a*, redness; CIE b*, yellowness

Means in the same row (a–c) with different letters are significantly different (*p* < 0.05)

All values are mean ± standard error

### Cooking yield and water holding capacity

Results of cooking yield and water holding capacity based on the level of *Magnolia denudata* extract are shown in Table 5. Cooking yield is an important factor that determines the productivity of a product. Weight loss occurs due to denaturation of meat proteins. Cooking yields of sausages added with *Magnolia denudata* extract ranged from 82.81% to 86.26%. Treatment groups showed significantly higher cooking yields than NC and PC groups (*P* < 0.05). Furthermore, the cooking yield tended to increase as the concentration of *Magnolia denudata* extract increased. Dietary fiber in food can enhance functional properties such as water retention and holding capacity (Seung, 1995). This suggested that increases in cooking yield and water holding capacity were due to the presence of insoluble dietary fibers such as cellulose and lignin in *Magnolia denudata* extract. A similar study by Lee et al. (2020b) on sausages made with pork and horseradish powder has reported that higher levels of dietary fiber from horseradish leaves can improve cooking yield.

**Table 5:** Cooking yield and WHC of chicken breast sausage formulated with various levels of *Magnolia denudate* extract

Traits(%)	NC	PC	T1	T2	T3
Cooking yield	78.97 ± 2.55^c^	78.83 ± 2.93^c^	82.81 ± 3.35^b^	85.42 ± 2.06^a^	86.26 ± 2.04^a^
WHC	72.44 ± 0.71^c^	85.97 ± 0.67^a^	75.09 ± 2.82^bc^	77.69 ± 0.54^b^	78.04 ± 2.59^b^

NC, no addition; PC, Ascorbic acid 0.1%; T1, *Magnolia denudate* extract 0.1%; T2, *Magnolia denudate* extract 0.15%; T3, *Magnolia denudate* extract 0.2%

Means in the same row (a–c) with different letters are significantly different (*p* < 0.05)

All values are mean ± standard error

In terms of water holding capacity, the PC group exhibited the highest value, likely because ascorbic acid, a strong antioxidant, inhibited protein oxidation and strengthened the structural stability of tissues, thus enhancing water-binding capacity (Ham et al., 2020; Moustafa et al., 2021). T1 and NC did not show a significant difference in water holding capacity (*P* > 0.05). However, T2 and T3 showed significantly higher water-holding capacity compared to the other treatment groups. This improvement in water holding capacity may be attributed to the high total phenolic content (TPC), total flavonoid content (TFC), and strong DPPH radical scavenging activity of the *Magnolia denudata* extract observed in this study, which can inhibit protein oxidation and preserve structural integrity, thereby enhancing water holding capacity. Therefore, it can be concluded that the addition of *Magnolia denudata* extract can increase both cooking yield and water holding capacity, with the group added with 0.2% of *Magnolia denudata* extract having the highest values of cooking yield and water holding capacity.

### pH

**Fig. 2:**
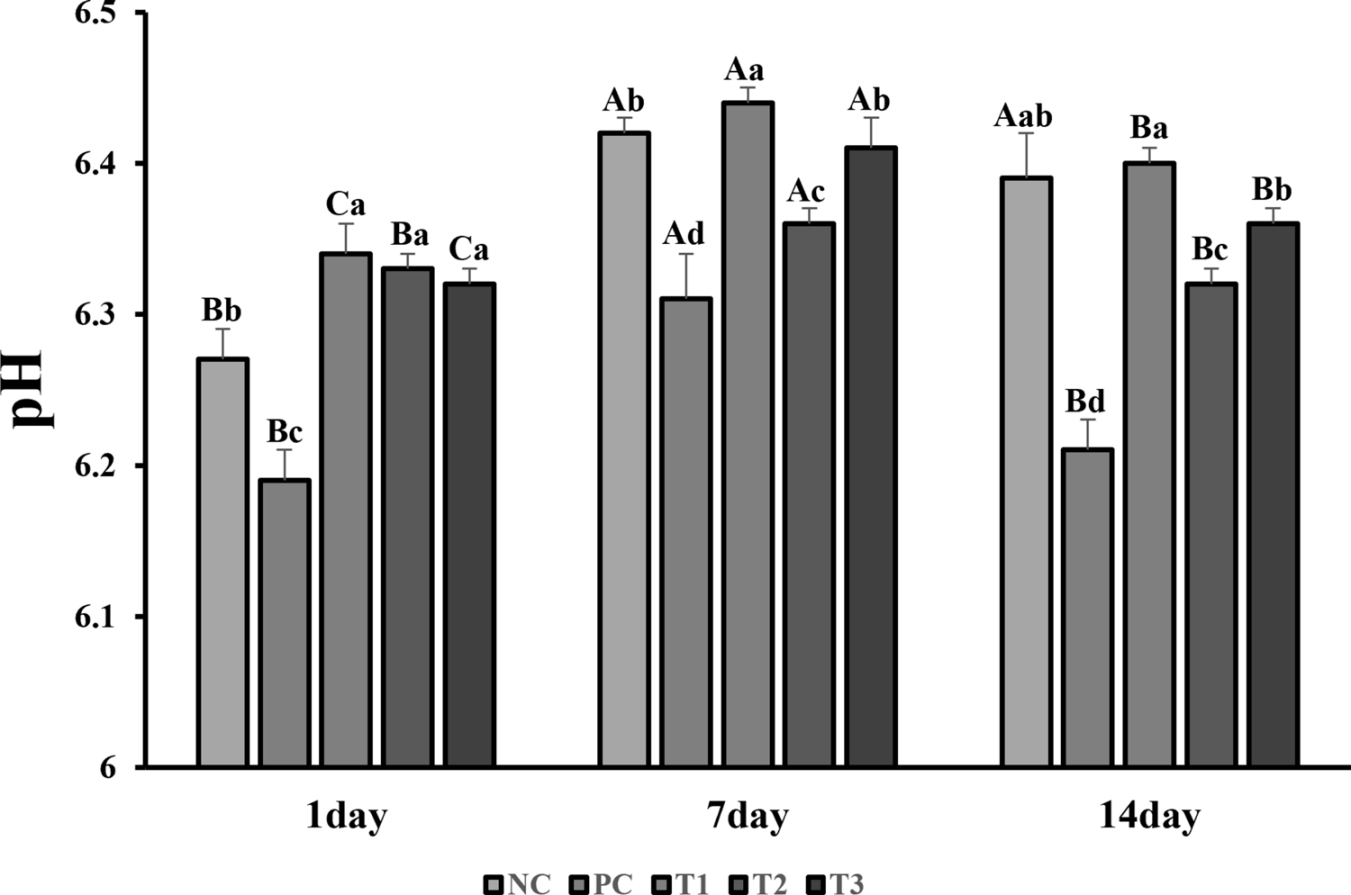
pH of chicken breast sausage formulated with various levels of *Magnolia denudate* extract during storage. NC, no addition; PC, Ascorbic acid 0.1%; T1, *Magnolia denudate* extract 0.1%; T2, *Magnolia denudate* extract 0.15%; T3, *Magnolia denudate* extract 0.2%. Means in the same row (a–d) and column (A–C) with different letters are significantly different (*p* < 0.05). All values are mean ± standard error

Figure 2 shows pH values according to the level of *Magnolia denudata* extract added and storage period. Treatment groups added with the *Magnolia denudata* extract exhibited significantly higher pH values than other groups (*p* < 0.05). This might be attributed to interactions of various compounds such as polyphenols and flavonoids with proteins, which can neutralize acidic groups (KISTI, 2013). In the case of the PC group, ascorbic acid acted as a redox agent that could lower the pH, resulting in significantly lower pH values compared to other groups. As the storage period increased, the pH increased in all treatment groups, with NC and PC groups showing greater increases in pH on day 7 compared to treatment groups. Kim et al. (2021) has suggested that this pH change occurs as microorganisms grow during refrigeration, leading to formation of alkaline substances such as ammonia and amines, which can then interact with water molecules and increase the pH of meat, similar to results of this study. Additionally, it has been reported that pH can increase due to breakdown of proteins and lipids caused by microbial growth, leading to formation of spoilage products (Kang et al., 2013). In all treatment groups, the pH increased on day 7 and then showed a slight decrease by day 14. This pattern was also observed in NC and PC. However, when pH values on day 1 and day 14 were compared, changes in pH for PC and T2 treatment groups were less significant. This suggests that high concentrations of antioxidant compounds might have inhibited changes of products.

### Thiobarbituric acid reactive substances (TBARS) measurement

**Fig. 3:**
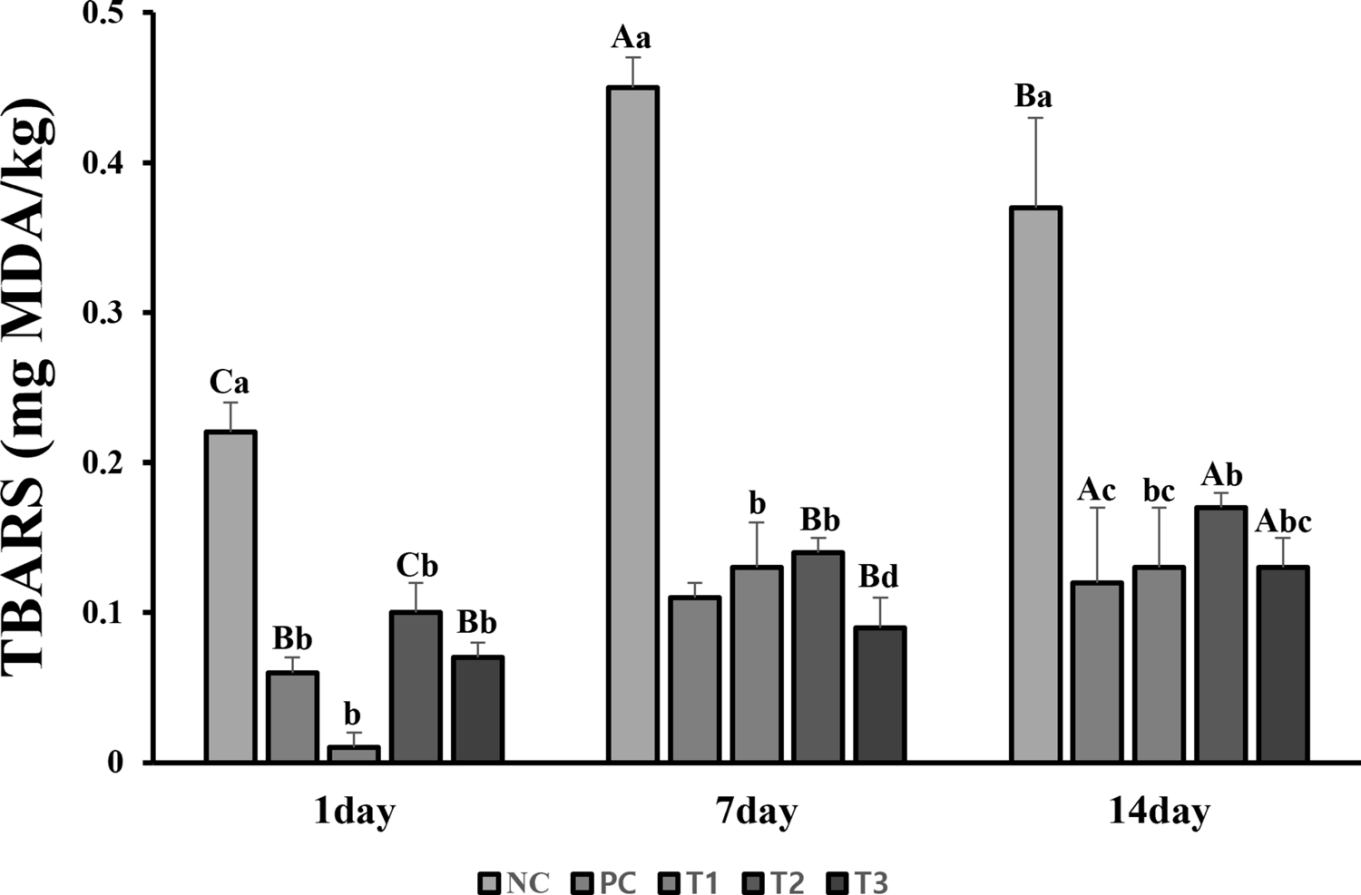
Thiobarbituric acid reactive substance of chicken breast sausage formulated with various levels of *Magnolia denudate* extract during storage periods (Unit: mg malondiadehyde/kg). NC, no addition; PC, Ascorbic acid 0.1%; T1, *Magnolia denudate* extract 0.1%; T2, *Magnolia denudate* extract 0.15%; T3, *Magnolia denudate* extract 0.2%. Means in the same row (a-d) and column (A–C) with different letters are significantly different (*p* < 0.05). All values are mean ± standard error

Analyzing thiobarbituric acid reactive substances (TBARS) is the most widely used method to measure fat oxidation in meat (Witte et al., 1970). Fat oxidation levels depending on the addition of *Magnolia denudata* extract and storage period are shown in Fig. 3. At week 0 of storage, the NC group exhibited a significantly higher fat oxidation value than other groups (*P* < 0.05), while PC, T1, T2, and T3 did not show significant difference in fat oxidation from each other (*P* > 0.05). This result suggests that absence of the *Magnolia denudata* extract in the NC group led to a lack of antioxidant compounds needed to prevent oxidation, resulting in higher fat oxidation levels.

At week 1 of storage, the NC group again showed a significantly higher fat oxidation than other groups (*P* < 0.05), while T3 exhibited a significantly lower value than other groups (*P* < 0.05). These results were similar to those found by Lee et al. (2020b) in sausages with a high content of antioxidant such as horseradish powder. T1 and T2 did not show significant difference from each other (*P* > 0.05). However, they had significantly higher values than the PC group (*P* < 0.05).

At week 2 of storage, the NC group showed significantly higher fat oxidation value than other groups (*P* < 0.05), while the PC group had a significantly lower value than T2, although it did not have a significantly different value from T1 or T3 (*P* > 0.05). Except for the NC group, all treatments including the PC group showed an increase in TBARS values as storage time progressed. However, the PC group did not exhibit significant differences over time (*P* > 0.05). T1 and T3 showed no significant difference between week 0 and week 1 (*P* > 0.05), although they did show significant differences at week 2 (*P* < 0.05). T2 showed a significant increase in TBARS value as storage time increased (*P* < 0.05).

Therefore, *Magnolia denudata* extract is effective in inhibiting fat oxidation during the storage of meat products. The T3 group exhibited the lowest TBARS values at week 1. This suggests that increasing the concentration of *Magnolia denudata* extract could more effectively inhibit fat oxidation, with a concentration of 2% being the most effective.

### Volatile basic nitrogen (VBN) measurement

**Fig. 4:**
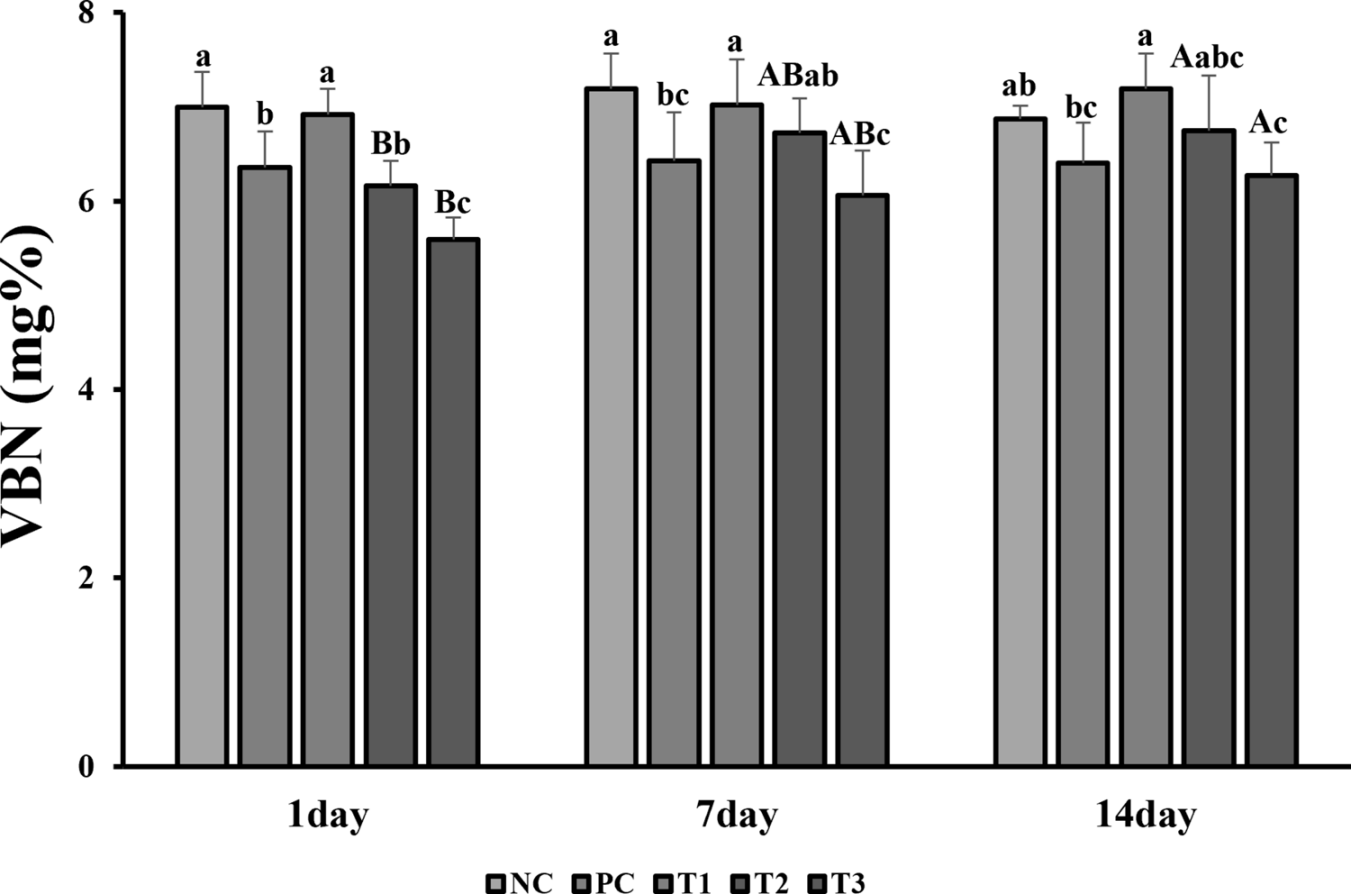
Volatile basic nitrogen of chicken breast sausage formulated with various levels of *Magnolia denudate* extract during storage periods. NC, no addition; PC, Ascorbic acid 0.1 T1, *Magnolia denudate* extract 0.1%; T2, *Magnolia denudate* extract 0.15%; T3, *Magnolia denudate* extract 0.2%. Means in the same row (a–c) and column (A-B) with different letters are significantly different (*p* < 0.05). All values are mean ± standard error

Volatile basic nitrogen (VBN) content was measured to determine the degree of protein degradation. VBN can serve as an indicator of freshness in meat and seafood. VBN levels according to the addition of *Magnolia denudata* extract and storage period are shown in Fig. 4. During all storage periods, T3 showed significantly lower VBN values than the NC group (*P* < 0.05). At week 0 of storage, T3 exhibited significantly lower than all other treatment groups (*P* < 0.05), while PC and T2 had significantly lower VBN values than NC and T1 (*P* < 0.05). At week 1, T3 showed significantly lower VBN values than NC and T1 (*P* < 0.05). However, it showed no significant difference from PC or T2 (*P* > 0.05). At week 2, PC and T3 showed significantly lower VBN values than T1 (*P* < 0.05), although no significant difference in VBN value was observed between NC and T2 (*P* > 0.05). Throughout all storage periods, NC, PC, and T1 showed no significant difference in VBN value (*P* > 0.05). T2 and T3 did not show significant difference in VBN value between week 0 and week 1. However, their VBN values were significantly higher in week 2 than in week 0 and week 1 (*P* < 0.05).

The increase in VBN level over time is believed to be due to breakdown of protein chains, leading to increases of non-protein nitrogen compounds such as amino acids, amines, ammonia, nucleic acids, and creatine (De Azevedo Gomes et al., 2003). According to Lee et al. (2020a), *Magnolia denudata* essence oil contains 77.07% 3-carene and 6.92% β-elemene, both of which have strong antimicrobial effects. In particular, 3-carene is effective against spoilage-causing bacteria such as *Brochothrix thermosphacta* and *Pseudomonas fluorescens*, while β-elemene is effective against foodborne pathogens such as *Staphylococcus aureus* (Zhu et al., 2013). Therefore, the addition of *Magnolia denudata* appears to effectively inhibit the growth of spoilage-causing bacteria, preventing the breakdown of fats and proteins in sausages, and improving their shelf life. Increasing the concentration of *Magnolia denudata* extract can more effectively inhibit protein degradation, with a 2% concentration showing the greatest efficacy.

### Sensory evaluation

Table 6 presents sensory characteristics of off-ordor, appearance, tenderness, juiciness, flavor, saltiness, bitterness, and overall preference based on addition level of *Magnolia denudata* extract. There were no significant differences in off-ordor, although the PC group received the highest score. As the concentration of *Magnolia denudata* extract increased, scores of treatment groups gradually decreased. In terms of appearance, the T1 group received the highest score, indicating a positive visual evaluation. This suggested that the concentration of *Magnolia denudata* extract did not significantly affect the appearance. Tenderness and juiciness were similar across all treatment groups, with T1 receiving slightly higher scores. For the flavor category, the PC group received the highest score, while treatment groups showed a decreasing trend for flavor scores as the concentration increased. For saltiness, the PC group had the highest score, although it showed no significant differences from other treatment groups. In terms of bitterness, T3 received the highest score, indicating that the bitterness increased as the concentration of *Magnolia denudata* extract increased. This suggests that high concentrations of antioxidant compounds might have contributed to the bitterness. Yu et al. (2018) has reported that high levels of total polyphenols and flavonoids in additives can contribute to bitterness, consistent with our findings.

**Table 6:** Sensory evaluation of chicken breast sausage formulated with various levels of *Magnolia denudate* extract

Traits	NC	PC	T1	T2	T3
Off-ordor	6.14 ± 2.19	7.14 ± 1.86	6.85 ± 1.86	5.86 ± 1.68	5.71 ± 2.19
Appearance	7.29 ± 1.60	7.14 ± 0.79	7.57 ± 0.79	6.71 ± 1.70	7.00 ± 1.60
Tenderness	5.71 ± 1.25	5.43 ± 1.57	5.86 ± 1.57	5.43 ± 2.07	5.14 ± 1.25
Juiciness	5.57 ± 1.51	5.86 ± 1.15	6.00 ± 1.15	5.86 ± 1.21	5.43 ± 1.51
Flavor	6.43 ± 1.81^ab^	7.43 ± 2.16^a^	5.00 ± 2.16^bc^	4.43 ± 1.81^bc^	3.71 ± 1.81^c^
Saltness	4.86 ± 2.12	5.71 ± 1.72	4.57 ± 1.72	4.72 ± 2.14	4.29 ± 2.12
Bitterness	1.14 ± 0.38^b^	1.14 ± 1.70^b^	5.29 ± 1.70^a^	5.86 ± 1.95^a^	6.57 ± 0.38^a^
Overall preference	6.93 ± 1.37^a^	7.86 ± 2.04^a^	4.86 ± 2.04^b^	4.57 ± 1.40^b^	3.71 ± 1.37^b^

Off-order, 1: Weak − 10: Strong; Appearance, 1: Bad − 10: Good; Tenderness, 1: Soft − 10: Hard; Juiciness, 1: Dry − 10: Juicy; Flavor, 1: Bad − 10: Good; Saltness, 1: Weak − 10: Strong; Bitterness, 1: Weak − 10: Strong; Overall preference, 1: Bad − 10: Good, Means in the same row (a–c) with different letters are significantly different (*p* < 0.05). All values are mean ± standard error

In the overall preference assessment, the PC group received the highest score, confirming its superior preference, while T3 received the lowest score, likely due to the strong bitterness perceived. T1 and T2 showed intermediate scores, with a trend of decreasing acceptability as the concentration increased. Results of this study showed that the concentration of *Magnolia denudata* extract had a significant impact on sensory characteristics of the product, with higher concentrations leading to stronger bitterness, which reduced overall preference. In contrast, the PC contained ascorbic acid maintained high preference, demonstrating both antioxidant and flavor improvement effects.

## Conclusion

This study analyzed antioxidant effects of *Magnolia denudata* extract and its impact on the quality and storage stability of chicken breast sausage during refrigerated storage. The *Magnolia denudata* extract exhibited a high antioxidant capacity of 44.48%. It contained 132.91 mg GAE/g of total polyphenols and 42.44 mg NE/g of total flavonoids. As the concentration of *Magnolia denudata* extract increased, the moisture content increased, while protein content decreased. This could be interpreted as a result of interactions between polyphenols and proteins, which can alter protein solubility and processing characteristics. Increases of cooking yield and water-holding capacity of sausages with higher *Magnolia denudata* extract concentrations suggest that antioxidant components can enhance emulsification stability and strengthen water-binding capacity by inhibiting protein oxidation. Smaller pH fluctuations in *Magnolia denudata* extract treated groups compared to the control group during storage might be due to antioxidant compounds inhibiting microbial activity and oxidation reactions, thereby delaying acidification. TBARS values were significantly lower in *Magnolia denudata* extract treated groups than in the control group throughout the storage period, with the rate of increase in TBARS during storage being slower in treated groups. This indicates that the antioxidant capacity of *Magnolia denudata* extract could inhibit fat oxidation and maintain the oxidative stability of sausages. VBN values were also significantly lower in the group treated with 0.2% *Magnolia denudata* extract, suggesting that antimicrobial effects of polyphenols and flavonoids suppressed protein degradation and reduced the formation of nitrogenous compounds. However, at a 0.2% concentration, increased bitterness led to a decrease in overall sensory preference. Although the 0.2% level was the most effective from a functional perspective, improving sensory acceptability will require the incorporation of additional ingredients or processing techniques to reduce bitterness. Future studies should investigate formulation strategies that maintain the functional benefits of *Magnolia denudata* extract while improving consumer preference.
